# Reliable computational quantification of liver fibrosis is compromised by inherent staining variation

**DOI:** 10.1002/cjp2.227

**Published:** 2021-06-02

**Authors:** Stuart Astbury, Jane I Grove, David A Dorward, Indra N Guha, Jonathan A Fallowfield, Timothy J Kendall

**Affiliations:** ^1^ NIHR Nottingham Biomedical Research Centre Nottingham University Hospitals NHS Trust and the University of Nottingham Nottingham UK; ^2^ Nottingham Digestive Diseases Centre, School of Medicine University of Nottingham Nottingham UK; ^3^ University of Edinburgh Centre for Inflammation Research University of Edinburgh Edinburgh UK; ^4^ Edinburgh Pathology University of Edinburgh Edinburgh UK

**Keywords:** liver fibrosis, histological scoring, artificial intelligence, digital pathology

## Abstract

Biopsy remains the gold‐standard measure for staging liver disease, both to inform prognosis and to assess the response to a given treatment. Semiquantitative scores such as the Ishak fibrosis score are used for evaluation. These scores are utilised in clinical trials, with the US Food and Drug Administration mandating particular scores as inclusion criteria for participants and using the change in score as evidence of treatment efficacy. There is an urgent need for improved, quantitative assessment of liver biopsies to detect small incremental changes in liver architecture over the course of a clinical trial. Artificial intelligence (AI) methods have been proposed as a way to increase the amount of information extracted from a biopsy and to potentially remove bias introduced by manual scoring. We have trained and evaluated an AI tool for measuring the amount of scarring in sections of picrosirius red‐stained liver. The AI methodology was compared with both manual scoring and widely available colour space thresholding. Four sequential sections from each case were stained on two separate occasions by two independent clinical laboratories using routine protocols to study the effect of inter‐ and intra‐laboratory staining variation on these tools. Finally, we compared these methods to second harmonic generation (SHG) imaging, a stain‐free quantitative measure of collagen. Although AI methods provided a modest improvement over simpler computer‐assisted measures, staining variation both within and between laboratories had a dramatic effect on quantitation, with manual assignment of scar proportion being the most consistent. Manual assessment also most strongly correlated with collagen measured by SHG. In conclusion, results suggest that computational measures of liver scarring from stained sections are compromised by inter‐ and intra‐laboratory staining. Stain‐free quantitative measurement using SHG avoids staining‐related variation and may prove more accurate in detecting small changes in scarring that may occur in therapeutic trials.

## Introduction

Histological assessment of liver scarring is a pivotal endpoint for determining efficacy of potential anti‐fibrotic therapies in clinical development. Conventional scoring systems encompass broad architectural distribution of fibrosis rather than reflecting only the amount of scar deposition [1] and rely on subjective interpretation by a trained pathologist. Therefore, subtle but potentially clinically significant improvements in histology that may predict endpoints such as portal hypertension and liver function may not be reliably captured.

Picrosirius red (PSR) staining is established as the most reliable method for visualising fibrosis in a liver biopsy, shows concordance with other measures of collagen deposition [2], and may show less staining variation than observed in trichrome staining or immunohistochemistry [1]. As measuring the intensity of a single colour on a slide is an extremely simple metric, it lends itself to computerised measurement that removes intra‐ and inter‐observer variation introduced by a pathologist score [3, 4]. This has led to the development of several computer‐aided methodologies, with varying degrees of success [5, 6]. These tools are often described as automated morphometry or collagen proportionate area (CPA) measurement and generally rely on tinctorial staining (Verhoeff's Van Gieson or PSR) to stain elastin and/or collagen fibres. Digital scans of these stained slides are then made and, by using a colour space threshold based on the hue, saturation, and brightness (HSB), a quantitative assessment of collagen or elastin over the entire section can be made. Such methods have been used to demonstrate differences between groups in translational or clinical research studies where staining can be undertaken in a tightly controlled, single/minimal batch manner by a single laboratory [5, 7].

The relative ease and declining cost of both acquiring and storing whole‐slide images mean that there is now a significant amount of histological data available that can be mined by machine learning algorithms. As opposed to CPA and associated techniques, machine learning enables the characterisation of ‘sub‐visual’ features of a slide – information that would not be consciously captured by a pathologist or simple computational methods [8, 9]. Machine learning methods can both be applied as a more sophisticated form of segmentation whereby an algorithm can be taught to distinguish features of a slide rather than using simple thresholds based on colour [10, 11], or be used to correlate complex histopathological features with clinical outcomes [12].

In addition to artificial intelligence (AI) methods, stain‐free second harmonic generation (SHG) and two‐photon excited fluorescence (TPEF) microscopy have been proposed as tools to enable a more accurate and objective assessment of a liver biopsy that is not influenced by staining quality [13]. SHG light is only generated by non‐centrosymmetric molecules such as collagen, therefore by exposing a tissue specimen to a laser and measuring the polarised light produced, an assessment of the amount and distribution of collagen can be made. SHG can be used in conjunction with TPEF microscopy, enabling visualisation of background liver tissue at the same time as collagen [14]. Currently unaffordable for routine diagnostic use, SHG/TPEF imaging can provide an accurate stain‐free quantitative measurement of fibrosis on a biopsy [15].

In this exploratory study, we have compared the performance of an AI methodology with simple thresholding and manual assessment in quantifying scar proportion in PSR‐stained sections of liver, alongside a stain‐free method of scar quantification. For widespread application in large clinical trials or routine clinical practice, the ideal method of scar quantitation from stained sections must be robust to staining variation both between and within laboratories where sections must be stained daily rather than as study‐specific batches. We have used sequential sections from the same blocks, stained on two separate occasions at two independent National Health Service (NHS) clinical pathology laboratories. In the absence of a ‘ground‐truth’, the performance of different methods of scar quantitation has been evaluated by the consistency of derived metrics of scar amount across the set of sequential stained sections, testing both inter‐ and intra‐laboratory effects, i.e. an optimal method would produce the same ‘result’ from each of the four sections from the same block stained on two separate occasions in two independent NHS laboratories. Finally, the stain‐based measurement methods of scar quantification were compared to stain‐free SHG/TPEF imaging, which gives a similar readout of the amount of collagen on a slide but is not subject to bias relating to either laboratory protocols or stain interpretation. Specifically, the measurement of fibrosis‐related parameters in liver tissue by SHG is highly reproducible when the test–retest performance has been evaluated [16].

The prevailing orthodoxy is that machine learning methods will provide a significant performance improvement over both simple colour space methods and human measurement, but we demonstrate significant challenges that must be overcome if AI methods are to be applied to histopathology in large multi‐centre studies and clinical practice.

## Materials and methods

### Human tissue acquisition and staining

Anonymised unstained formalin‐fixed paraffin‐embedded sections from 20 cirrhotic explant livers (four cases each with alcoholic liver disease, non‐alcoholic fatty liver disease, chronic hepatitis C virus infection, primary sclerosing cholangitis, and primary biliary cholangitis as the stated primary aetiology) were provided after approval by the Lothian NRS Human Annotated Bioresource with permission granted under authority from the East of Scotland Research Ethics Service REC 1, reference 15/ES/0094.

From each block, initial five adjacent 5 μm sections were cut for staining in Nottingham and Edinburgh. A single section from each case was PSR stained according to standard local protocols within two CPA UK‐accredited NHS pathology laboratories – Nottingham University Hospitals NHS Trust Queen's Medical Centre Pathology Department and NHS Lothian Department of Laboratory Medicine at the Royal Infirmary of Edinburgh (see Supplementary materials and methods). To assess intra‐laboratory variation, staining on each case was repeated at both laboratories 6 months later using another section of the initial set from the same block, generating four stained sets of slides in total. Finally, further sections were cut from the same blocks and stained in Nottingham, where the standard staining protocol was unchanged, within 1 week to further evaluate intra‐laboratory staining variation; the standard protocol for PSR staining in Edinburgh had changed after the two rounds of staining, so an additional round of staining was not undertaken.

### Image acquisition and processing

Stained sections were scanned using identical NanoZoomer scanners (Hamamatsu Photonics, Shizuoka, Japan) at ×20 magnification. The raw scanned .ndpi whole‐slide images were split by ndpisplit [17] into ×5 magnification 1,000 × 1,000 pixel tiles in TIFF format. As the scans contained the entire slide, including areas not containing tissue, simple thresholding was used to isolate the tissue from each tile and discard empty space and debris contained in each scan. The script used to isolate tissue is included in supplementary material, File S1. Overview images at ×1.25 were exported from the raw .ndpi file and are available from the University of Nottingham Research Data Repository (https://rdmc.nottingham.ac.uk/handle/internal/9133).

### Manual scoring

All livers were cirrhotic, so the application of traditional ordinal scores of architecture provided no inter‐case discrimination. Instead, whole‐slide images of each section were stripped of all identifiers and randomly numbered. Four participants (two qualified pathologists and two non‐clinical researchers) provided their assessment of the percentage of tissue on each slide that was PSR positive, scoring each batch of 20 slides with a ‘washout’ period of at least 1 day in between different, randomly ordered batches.

### HSB colour space scar quantification

Each background cleaned tile was classified by two separate HSB colour space thresholds to calculate the number of pixels representing total tissue and the number of PSR‐positive pixels. The threshold values to determine PSR‐positive pixels were derived by selecting positive pixels within a representative tile and then testing on representative tiles from all cases, adjusting and iterating by‐eye until the most consistent thresholding was achieved. A single set of threshold values was applied to tiles from all stained image sets.

### Classifier development in WEKA

Following pre‐processing described above, the Waikato Environment for Knowledge Analysis (WEKA) [18], an open‐source Java‐based tool available as a plugin within Fiji [19], was used to build a PSR classifier. Tiles were randomly selected from the data set and used to train the classifier. Classes were defined as ‘Space’ (empty space surrounding extracted portions of tissue), ‘Lumen’, ‘PSR positive’, and ‘Tissue’. Areas of each tile were selected using a graphics tablet and manually defined as one of the four classes. WEKA was set up to use mean, minimum, maximum, median, and variance as training features for the selected pixels. The balance classes setting was used to account for differences in the amount of training data used for each of the four classes. Once the areas of each tile were defined, these training data were used by WEKA to extrapolate across the entire tile, giving an image segmented into four colours based on the defined classes. Training was then repeated on each tile until it was segmented into the four classes accurately, as judged by a pathologist. Training was then continued using at least one tile from each of the 20 slides in the study. Following training, a script was used to apply the classifier to every tile in the study and count the number of pixels in each class. This script is included in supplementary material, File S2. PSR positivity was defined as the number of PSR‐positive pixels divided by total PSR‐positive pixels + tissue + lumen and expressed as a percentage.

Individual WEKA classifiers (WEKA_i) specific to each single set of images were trained by using only tiles from that stained image set. For combination WEKA classifiers (WEKA_c1 and c2), classifiers were trained using tiles drawn from all stained image sets.

### SHG/TPEF imaging

SHG/TPEF imaging was carried out by Histoindex Pte Ltd (Singapore) using an unstained section from each of the 20 cases, at ×20 magnification. The raw SHG percentage (a measure of the amount of collagen) and qFibrosis, a score taking into account both the amount and distribution of collagen [20], were correlated with the other stain‐based scoring methods.

### Statistical comparisons between methods

**Figure 1:**
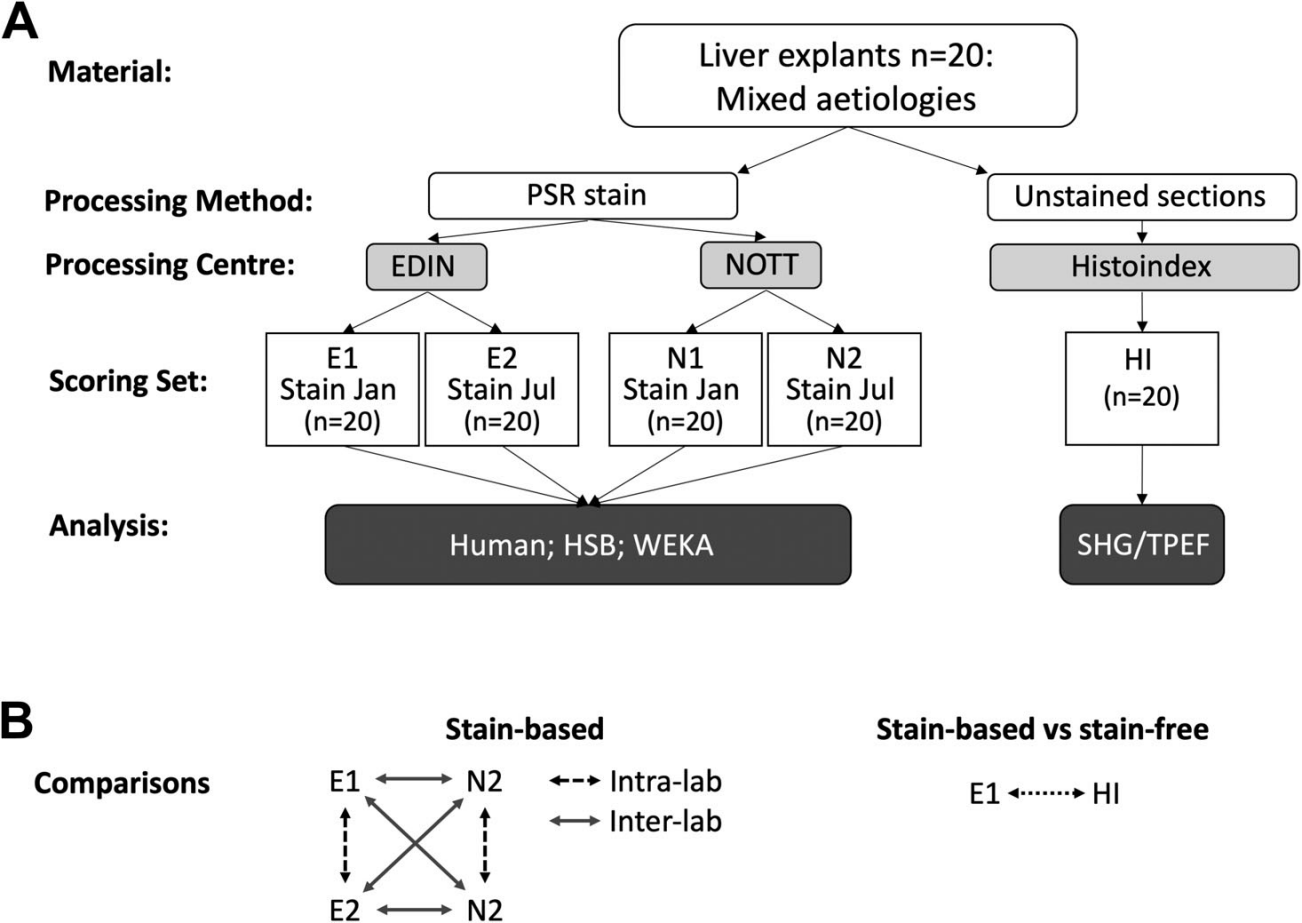
Outline of the study design. (A) Twenty explants were PSR stained at two different laboratories (Edinburgh and Nottingham), with each laboratory staining in two batches of 6 months apart, giving four sets of 20 slides each (E1, E2, N1, and N2). The stained slides were then scored using three different methods (human, HSB, and WEKA). A fifth set of slides were sectioned and left unstained for SHG/TPEF imaging. (B) Each stained set of slides gives six measurement pairs that can be compared to assess inter‐ and intra‐laboratory variation with each scoring method. A single set of stained slides (E1) was used as the comparator with the stain‐free SHG/TPEF set.

The combination of metrics from four stained sections of the initial set from each case, Edinburgh 1 and 2 (E1 and E2) and Nottingham 1 and 2 (N1 and N2) from three different measurement approaches resulted in six pairs of observations used to compare the different methods (Figure 1). Scores derived from the unstained set (SHG/TPEF) were correlated with scores from each measurement method on the E1‐stained set. Spearman correlation coefficients were calculated for each pair of observations. Metrics from the freshly recut sections stained in Nottingham (rN3) were compared with those derived from N1 and N2 alone. Scores for each section using each of the measurement methods are included in supplementary material, File S3.

## Results

### Inter‐ and intra‐laboratory staining variation significantly reduces consistent PSR quantification using computational methods

**Figure 2:**
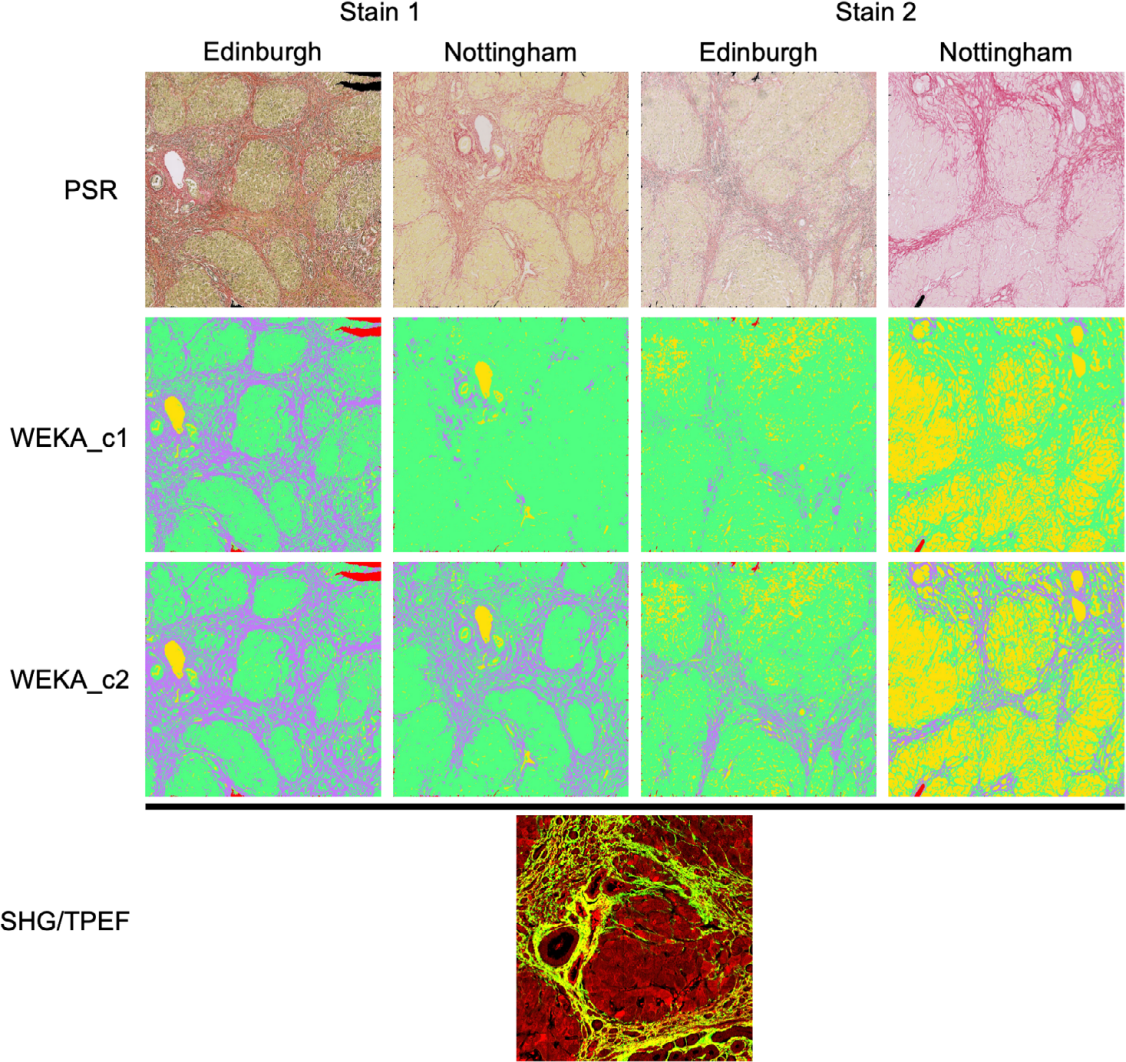
Representative illustration of intra‐ and inter‐laboratory PSR staining differences and the effect on segmentation using HSB and WEKA classifiers and comparison to SHG/TPEF imaging. WEKA features are coloured as purple = PSR positivity, yellow = lumen, green = tissue, and red = blank space. WEKA_c1: WEKA classifier trained on sections from both laboratories. WEKA_c2: WEKA classifier c1 with further targeted training on sections with greater than 2× divergence in PSR quantification between stain pairs. SHG/TPEF image is coloured as collagen in green/yellow and parenchyma in red.

Slides were stained at two centres (January 2018), followed by staining of a second set 6 months later at both centres (July 2018), using the next sections from each FFPE block that were cut at the same time at the start of the study to allow evaluation of intra‐laboratory staining variation in a routine, real‐world clinical laboratory context. We observed substantial qualitative differences in the PSR colour and intensity in each batch stained, even when comparing slides stained at the same centre (Figure 2).

**Figure 3:**
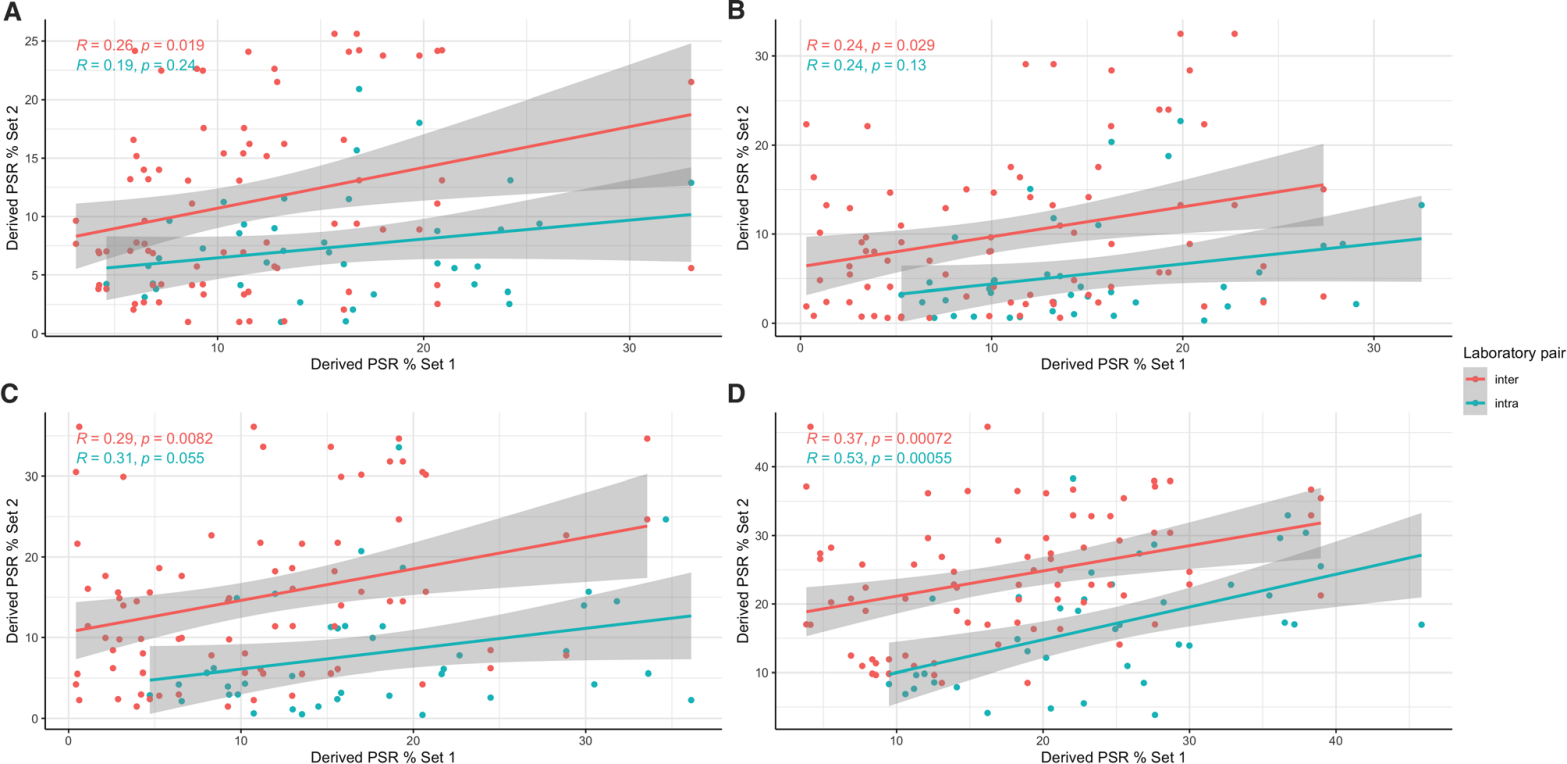
Scatterplots comparing all possible PSR stain pairs specified in Figure 1 between and within the two laboratories, all correlations are Spearman's rho. (A) HSB. (B) WEKA_i: WEKA classifier trained only on sections from either Edinburgh or Nottingham laboratories individually. (C) WEKA_c1: WEKA classifier trained on sections from both laboratories. (D) WEKA_c2: WEKA classifier c1 with further targeted training on sections with greater than 2× divergence in PSR quantification between stain pairs.

Measurement of scar proportion using a single HSB colour space threshold applied to all staining sets (E1, E2, N1, and N2) showed large differences between derived values of PSR percentage for each given case. The Spearman's rank correlation of values was poor both for inter‐laboratory (*ρ* = 0.26) and intra‐laboratory (*ρ* = 0.19) stain pairs (Figure 3A).

A WEKA classifier was trained using training data from and applied to each individual set of PSR‐stained slides (E1, E2, N1, and N2) in isolation (WEKA_i). The classifiers trained on and then applied to each individual set of stained slides (WEKA_i) produced no increase in consistency compared with the simple colour space thresholding method. Spearman's correlation coefficients were similarly low for both intra‐ (*ρ* = 0.24) and inter‐laboratory stain sets (Figure 3B).

A second unified WEKA classifier was trained with images from both sites (WEKA_c1) and applied to all cases. Unified training marginally increased the consistency of the classifier, with slightly increased Spearman's correlation coefficients for both intra‐ (*ρ* = 0.31) and inter‐laboratory (*ρ* = 0.29) stain sets (Figure 3C).

To explore the potential of using further training to iteratively improve classifier accuracy, sections that produced especially divergent results (inter‐laboratory pairs displaying over 2× divergence in scoring) were used to further train an improved combined classifier (WEKA_c2). This targeted training led to further improvements in classifier consistency across all images, and increased Spearman's correlation coefficient for both intra‐ (*ρ* = 0.53) and inter‐laboratory (*ρ* = 0.37) stain sets (Figure 3D).

By comparing the derived scar proportion (PSR‐positive percentage of tissue) using each classifier applied to each set of stained images (E1, E2, N1, and N2), it is evident that both inter‐ and intra‐ laboratory staining differences have a significant impact on classification (Figures 2 and 3). Most importantly, the change in colour of the PSR stain led to a significant reduction in the number of pixels classified as PSR positive by certain classifiers (Figure 2). Some of this was corrected with further training (Figure 2, WEKA_c2). This staining variation also led to significant misclassification of non‐PSR‐positive tissue, in particular, the incorrect classification of liver tissue as vessel lumen (Figure 2, Nottingham stain 2).

To explore whether the duration between block sectioning and staining could account for some of the intra‐laboratory staining variation, a freshly cut set of sections from the same blocks were stained in Nottingham (rN3), where the PSR staining protocol remained unchanged, in February 2021. The derived scar proportion using the WEKA_c2 classifier from rN3 most closely correlated with the derived values from N1, suggesting that the duration of time between section preparation and staining was responsible for a proportion of the intra‐laboratory variation in PSR staining (see supplementary material, Figure S1).

### Human assessment of scar proportion is significantly more consistent than computational methods

Although the current gold standard is ordinal scoring of architecture by a pathologist, the scales are crude. For example, all cases in this study were cirrhotic and so would be assigned the same score in any system used. Computational analysis is purported to outperform a human observer in determining the absolute amount of a feature of interest, such as the percentage of tissue that is PSR positive, and so such estimates by an observer are rarely used.

However, we tested whether such confidence in computational methods, or more correctly scepticism about the performance of human observers, was valid. Four observers, two consultant pathologists and two research scientists, were asked to give an estimate of the percentage of the tissue that was PSR positive from randomly ordered low‐power thumbnail images of each stain set (E1, E2, N1, and N2), repeating the process on renumbered image sets at least 24 h later. No prior training for the task was provided.

**Figure 4:**
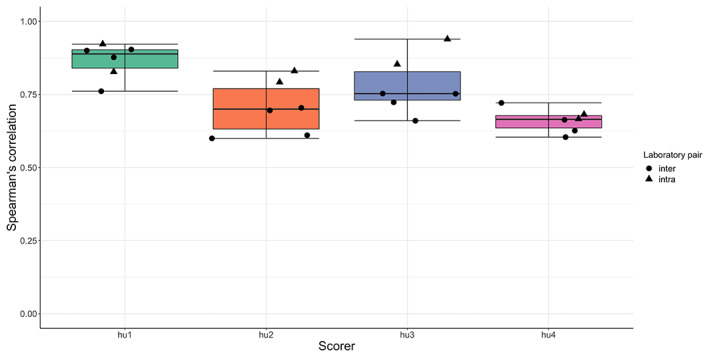
Spearman correlations of all slide pairs scored by four humans on four separate occasions by volunteers (two trained pathologists [hu1 and hu2] and two non‐clinical researchers [hu3 and hu4]).

**Figure 5:**
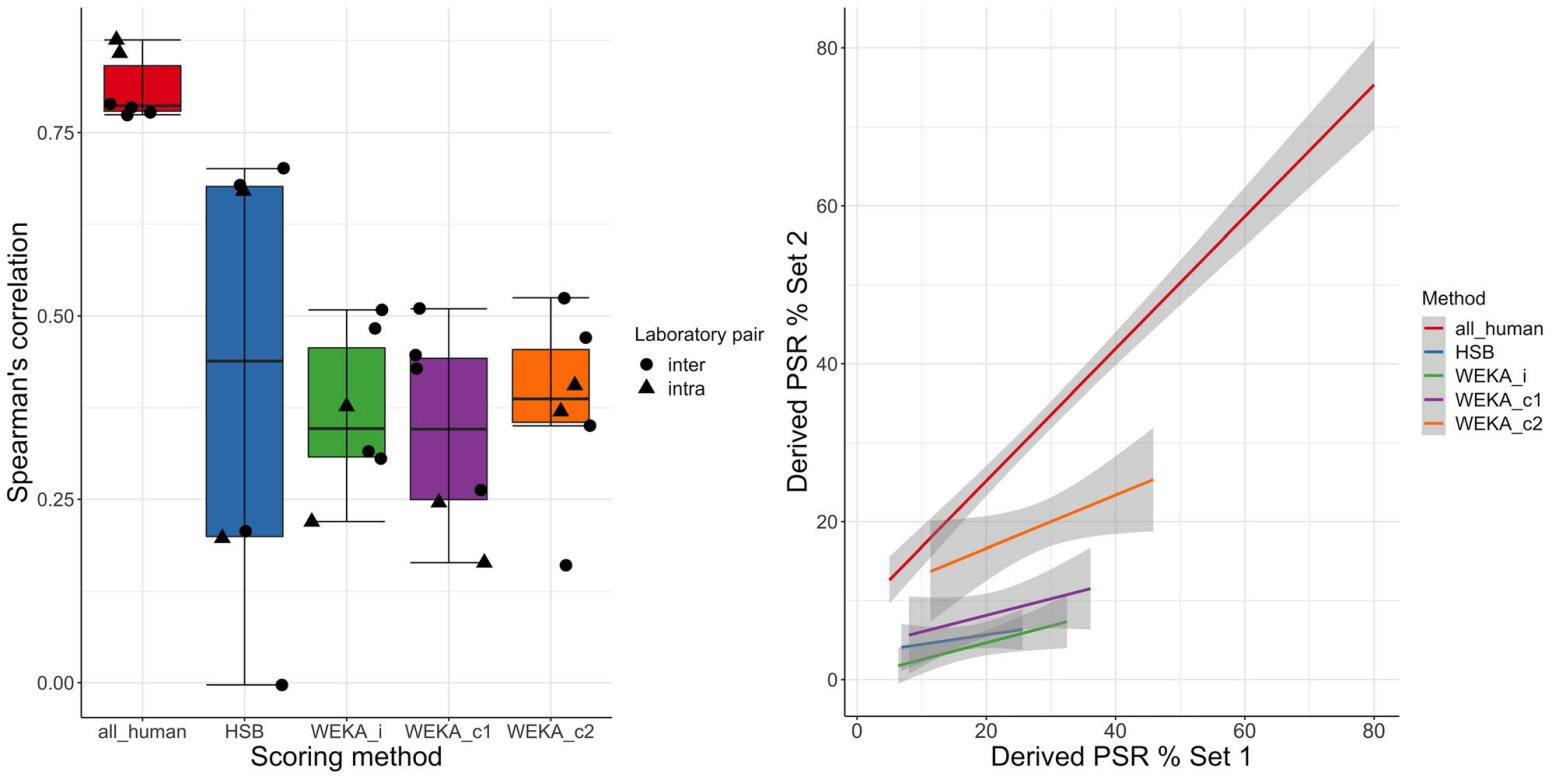
Boxplot of Spearman correlations for every measurement method (left) and combined scatterplot of scores for each measurement method (right).

Against all expectations, there was much greater consistency of given scar proportion across the stain sets for each observer alone compared with any computational method (Figures 4 and 5), regardless of whether the scorer was a pathologist (hu1 and hu2) or non‐clinical researcher (hu3 and hu4). This indicates that an observer is much more able to compensate for variations in staining than any computational method.

### Comparison of computational methods on stained sections with SHG/TPEF imaging

**Figure 6:**
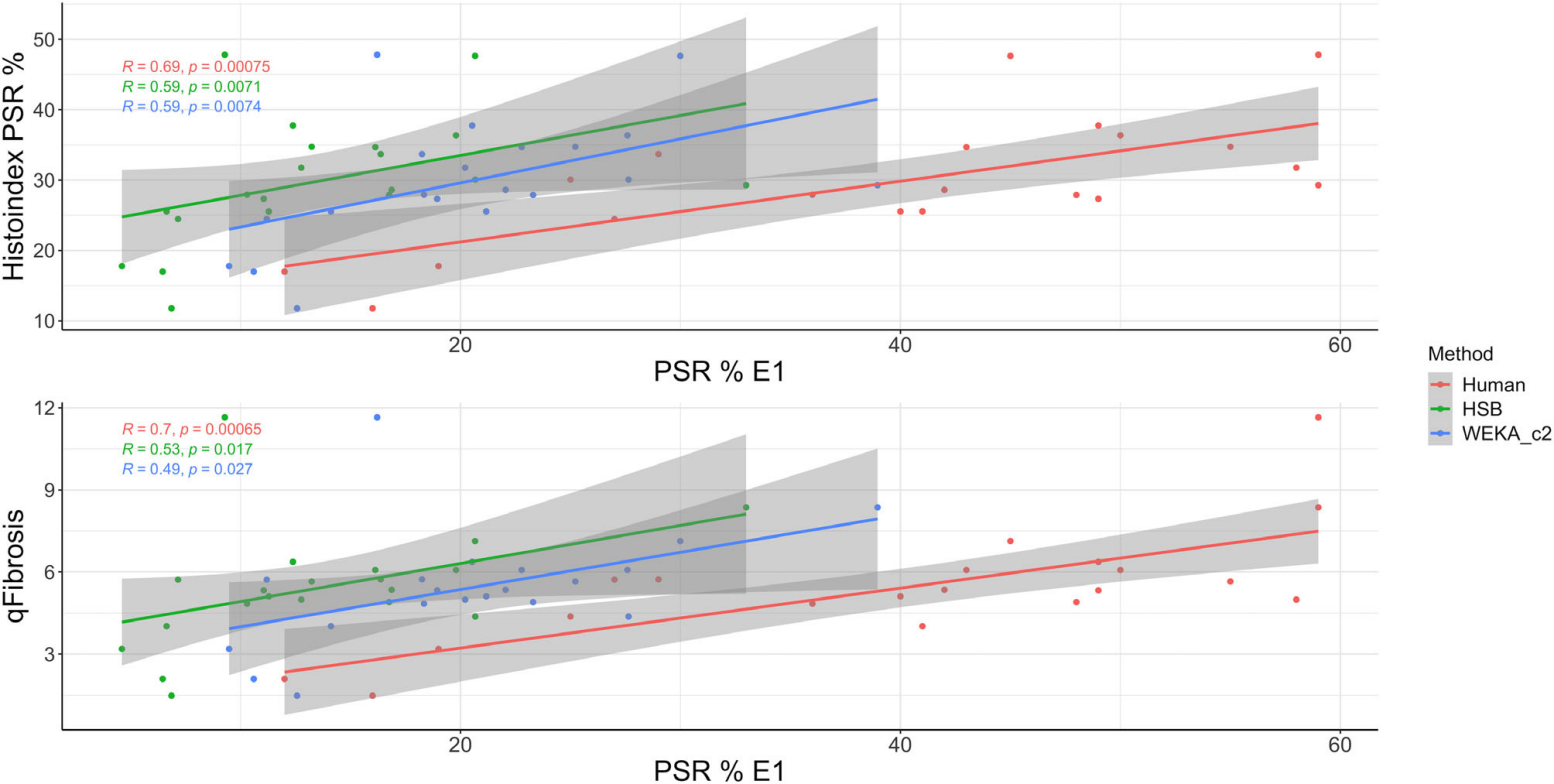
Using a single stained set of slides (E1), the stain‐based scoring methods were compared to percentage SHG measured using stain‐free SHG/TPEF imaging and the qFibrosis index derived from the measured parameters. WEKA_c2: WEKA classifier c1 with further targeted training on sections with greater than 2× divergence in PSR quantification between stain pairs.

Having assessed an AI‐based method against existing methods of stain‐based measurement, we then compared these methods to commercially available, stain‐free SHG/TPEF imaging (Figures 1 and 2). HSB colour space thresholding, the most consistent AI classifier (WEKA_c2), and the most consistent human scorer (hu1) for readouts on the E1 set, based on median correlation between all stain pairs, were used as comparators with SHG. Scores were compared against both the raw SHG value (expressed as a percentage of the total amount of tissue scanned) and the qFibrosis score, which adjusts the SHG percentage based on its distribution across the scanned section. In both instances, human scoring gave the strongest correlation with SHG/TPEF quantification (Figure 6).

## Discussion

In 2015, a report commissioned by the UK government's Minister for Digital and Culture outlined potential benefits and opportunities of AI and machine learning tools, including how these could be applied to health care [21]. In 2019, £250 m was invested in a National Artificial Intelligence Lab, to be based within NHSX [22]. Thus, there is a clear drive among both politicians and the largest technology companies in the world to apply AI methods wherever possible in medicine.

In the research setting, large, multi‐centre trials using liver fibrosis as a primary efficacy endpoint currently rely on ordinal scores such as the non‐alcoholic fatty liver disease (NAFLD) activity score or Ishak fibrosis stage. Even when best practices are followed (central review by more than one pathologist, central staining if practical, and consistency in biopsy technique) [23], there is potentially a significant amount of information lost through the use of these ordinal scoring systems. Ideally, computational methods including those using AI would provide a way to both extract information from liver biopsies that is not represented by ordinal scoring, whilst also removing the subjectivity inherent to the process of scoring.

However, there are several challenges that machine learning‐based tools need to overcome if they are to be utilised in a clinical or research setting, including, but not limited to, a reliance on retrospective rather than prospective studies, the lack of standardisation to enable comparison between different AI tools, and the ‘AI‐chasm’, a term defining the gulf between reported accuracy measurements of a given machine learning tool during development and its actual diagnostic efficacy when used in the field [24]. The AI‐chasm problem was illustrated by a Google‐developed tool for detecting diabetic retinopathy using scanned images of retinas, which displayed high accuracy during training but was significantly affected by inter‐site variation when applied in a live setting [25]. A systematic review of AI tools published in 2019 highlighted that few studies make direct comparisons between a tool and healthcare professionals, and even fewer use external validation. In studies where external validation was compared to internal validation, internal validation was shown to overestimate the effectiveness of AI compared to healthcare professionals [26]. Using readily accessible, open‐source machine learning tools to measure a simple histological feature, we have demonstrated that staining variation both within and between laboratories will pose significant challenges if these tools are to be applied even in the tightly controlled environment of multi‐centre studies.

As there is a lack of an established gold standard for measuring the amount of scarring in a liver biopsy, we have used consistency of the derived scar proportion across the four sets of stained sections as the metric to assess the performance of each method. Methods that are more robust to staining variation will produce more consistent values and give a tighter correlation between stain pairs.

Our study demonstrates that a trained AI‐based method does increase consistency compared with simple colour space thresholding, an increase in performance that is enhanced by further training. As expected, this increase in consistency was higher between intra‐laboratory stain pairs, with protocol and environmental differences between laboratories more likely to produce significant changes in staining compared to reagent changes within a single laboratory. However, there was still considerable residual inconsistency in the calculated scar proportion and human observers were easily able to outperform these methods, despite the task putatively favouring computational methods. As observed for the computer‐based scoring methods, the human scores also showed a slightly higher consistency between intra‐laboratory pairs compared to inter‐laboratory pairs (Figure 4).

The age of a histological section is known to affect a variety of stains [27], therefore staining was repeated in the Nottingham laboratory on a freshly cut set of sections (rN3) ensuring 1 week between sectioning and staining. Classifying with the most consistent AI classifier (WEKA_c2) produced significant correlations between both the newly stained set rN3 and N1 and N2 stains, with a closer correlation observed between N1 sections and the rN3 set (see supplementary material, Figure S1). As the time between sectioning and staining was greater in N2 compared to N1 sections, this indicates that section age may contribute to intra‐laboratory variation if a standard interval between sectioning and staining is not used. The other sources of intra‐laboratory staining variation can only be speculated upon, but may include inter‐operator differences in the application of hand‐staining protocols, the age of reagents, and seasonal and diurnal variation in the laboratory air and water temperatures.

We present this not to suggest that by‐eye estimations of scarring should be used but to highlight that staining variation is an inevitable factor in real‐world laboratories. Whilst iterative training will undoubtedly increase the consistency of methods used to assess scarring in stained sections and more sophisticated tools are in development to effectively allow ‘normalisation’ between multiple staining sites to attempt to account for the variation introduced [28, 29], stain‐free methods that are not affected by such variation should be considered. Existing quality control efforts in histopathology focus on maintaining consistency particularly with regard to immunohistochemistry, where there is a greater variation in staining protocols and reagents compared to tinctorial staining. This study indicates that similar efforts (protocol standardisation, the use of tissue controls, and colour calibration) would be required if AI‐assisted scoring of tinctorial stains is to be applied widely.

SHG/TPEF imaging has been proposed as a gold standard for the measurement of liver fibrosis [30], particularly in the context of clinical trials, where quantifying potentially small changes through the course of a study is required. Our comparison of each of the stain‐based methods of collagen quantification with both raw SHG percentage and the qFibrosis score demonstrated that human scoring is the most strongly correlated, again suggesting AI methods are more vulnerable to inter‐ and intra‐laboratory staining variation than humans. The common advantage of both humans and SHG/TPEF is their ability to consider beyond colour quantification. Both can utilise information from the tissue architecture and ‘landscape’ of the scar either unconsciously by humans or using feature recognition processes that are not biased by staining to quantify based on different aspects of fibrosis.

The study is limited in the type of specimen and the stain assessed. Only sections from explant livers were used. Whilst this type of specimen in only encountered by laboratories at liver transplant centres, it was chosen because the available tissue for research was abundant with no risk of exhausting the blocks. Whilst the use of explants meant that the study was limited to cirrhotic livers, rather than representing the full spectrum of disease stage, there is no clear reason that the findings cannot be extrapolated to PSR‐stained sections with any amount of fibrosis. The examination of a set of cases where ‘gold‐standard’ ordinal scoring of fibrosis is unambiguously non‐informative (i.e. all cases are assigned the same score under any pan‐aetiology or aetiology‐specific scoring system) serves to illustrate the potential value for formal computational quantification. Finally, only PSR‐stained sections were examined. We would suggest that whilst the sources and extent of staining variation will vary depending on the specific stain, the susceptibility of computational methods of feature quantification in stained sections to such variation should always be evaluated where studies use anything other than self‐contained, single batch staining.

In conclusion, we demonstrate that computational tools are not yet able to satisfactorily compensate for differences in tissue staining both between and within laboratories. The results here suggest that caution should be exercised when applying such methods to stain‐based quantification in histopathology, particularly in large multi‐centre studies, without applying extremely rigorous standardisation between staining centres.

## Author contributions statement

SA collected the data, analysed the data, and prepared and revised the manuscript. JIG obtained funding, collected the data, and prepared and revised the manuscript. DAD collected the data and revised the manuscript. ING obtained funding, designed the study, and revised the manuscript. JAF designed the study and revised the manuscript. TJK designed the study, collected the data, analysed the data, and prepared and revised the manuscript.

## Supporting information


Supplementary materials and methods
**Figure S1.** The effect of section age

**File S1.** Script in ImageJ Macro Language to use in FIJI

**File S2.** Script in ImageJ Macro Language to use in FIJI

**File S3.** Excel file containing measurements using all stain‐based methods (HSB, WEKA, and manual scoring) and stain‐free methods (SHG % and qFibrosis score)

## Data Availability

Data and scripts are available in supplementary information.
