# Supplementary material for: Reliable computational quantification of liver fibrosis is compromised by inherent staining variation
Source: J Pathol Clin Res. 2021 Jun 2;7(5):471–81. doi: 10.1002/cjp2.227 (PMC8363922; doi:10.1002/cjp2.227)
Supplement: Supplementary file 1 — Supplementary materials and methods Figure S1. The effect of section age [file CJP2-7-471-s003.docx]

# Reliable computational quantification of liver fibrosis is compromised by inherent staining variation

## S Astbury *et al*. *J Pathol Clin Res* DOI: 10.1002.cjp2.227

## **Supplementary Material**

Files S1-S3 are provided separately

**Supplementary Materials and Methods**

**Figure S1.** The effect of section age

**File S1.** select_tissue_fiji_script.ijm – script in ImageJ Macro Language to use in FIJI; this applies thresholding to isolate tissue from a scanned image and remove blank space and debris.

**File S2.** WEKA_classification_fiji_macro.ijm – script in ImageJ Macro Language to use in FIJI; this applies a trained WEKA classifier to a folder of images and outputs number of pixels for each defined class in each image as a .txt file.

**File S3.** all_measurements.xlsx – Excel file containing measurements using all stain-based methods (HSB, WEKA and manual scoring) and stain-free methods (SHG % and qFibrosis score).

### **Supplementary Materials and Methods**

### **PSR staining protocol, Edinburgh**

1. Take sections to water.
2. 5 min celestine blue.
3. 15 s immerse in running water.
4. 4 min haematoxylin.
5. 10 s immerse in running water.
6. 30 s Scott’s tap water.
7. 15 s immerse in running water.
8. 5 min picric acid.
9. 5 min picro-sirius red solution (1 ml 1% aqueous sirius red F 3B, 10 ml saturated aqueous picric acid).
10. Rinse in 70% industrial denatured alcohol.
11. Dehydrate, clear and mount section.

### **PSR staining protocol, Nottingham**

1. Take sections to water.
2. 60 min Picro Sirius Red (0.1 g Sirius red, 100 ml saturated aqueous picric acid, revalidate monthly, 6-month shelf life).
3. Blot sections on fibre free blotting card.
4. Rinse rapidly in industrial methylated sprit.
5. Dehydrate, clear and mount section.


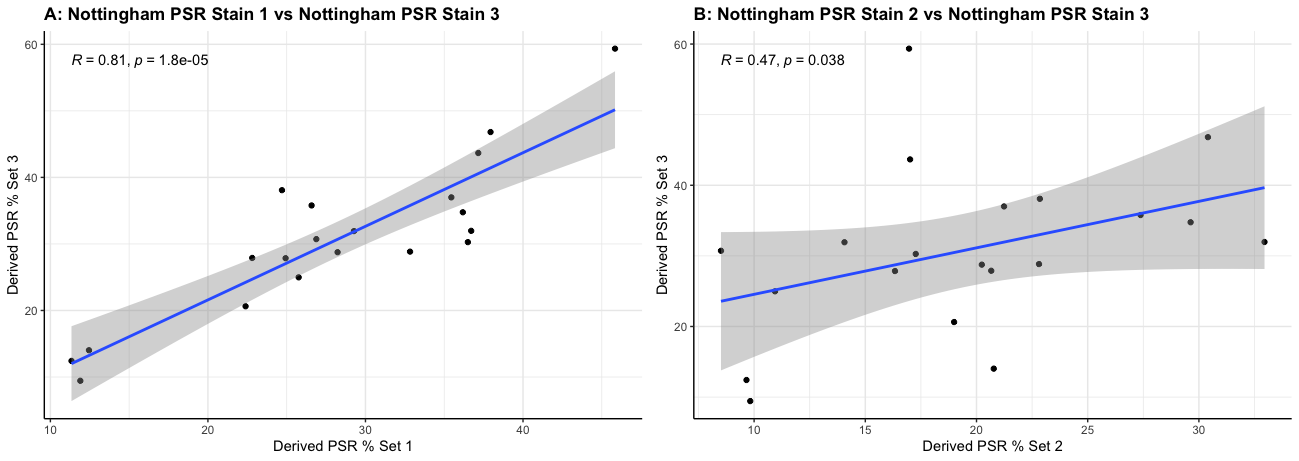


**Figure S1.** To assess the effect of section age, stain sets N1 and N2 were compared to a third set of freshly cut sections (rN3), stained following the same protocol with 1 week between sectioning and staining. This third stain set was classified using the WEKA_c2 classifier without further training and compared to scores from the same classifier on N1 (panel A) and N2 (panel B) slides. Correlation coefficients are Spearman’s rho.
